# Heterodimers reign in the embryo

**DOI:** 10.7554/eLife.33682

**Published:** 2017-12-27

**Authors:** Benjamin Tajer, Mary C Mullins

**Affiliations:** Department of Cell and Developmental BiologyUniversity of Pennsylvania Perelman School of MedicinePhiladelphiaUnited States

**Keywords:** axis formation, left-right patterning, Nodal signaling, Gdf3, mesoderm, endoderm, Zebrafish

## Abstract

Experiments by three independent groups on zebrafish have clarified the role of two signaling factors, Nodal and Gdf3, during the early stages of development

**Related research article** Montague TG, Schier AF. 2017. Vg1-Nodal heterodimers are the endogenous inducers of mesendoderm. *eLife* **6**:e28183. doi: 10.7554/eLife.28183

**Related research article** Bisgrove BW, Su YC, Yost HJ. 2017. Maternal Gdf3 is an obligatory cofactor in Nodal signaling for embryonic axis formation in zebrafish. *eLife* **6**:e28534. doi: 10.7554/eLife.28534

**Related research article** Pelliccia JL, Jindal GA, Burdine RD. 2017. Gdf3 is required for robust Nodal signaling during germ layer formation and left-right patterning. *eLife* **6**:e28635. doi: 10.7554/eLife.28635

The formation of the basic embryonic body plan of all animals depends on signaling factors that cause naïve cells to differentiate into cells with a wide range of potential fates. Key among the earliest such signals in vertebrates is a signaling factor called Nodal, which specifies the mesoderm (the tissue that goes on to become muscle and bone) and the endoderm (which is the precursor to the liver, stomach and other internal organs). Additionally, Nodal signaling is essential to instructing organs, such as the heart and liver, to form on either the left or right side of the embryo (reviewed in Zinski et al., 2017).

Like Nodal, Gdf3 is a member of the TGFß family of signaling factors, but even though it was discovered more than 30 years ago, much less is known about Gdf3 (which is also called Gdf1 or Vg1). However, we do know that Gdf3 and Nodal are expressed in the same tissues, and that they require the same signaling pathway components (Cheng et al., 2003; Zinski et al., 2017)), which has fuelled speculation that they work together, perhaps by forming a heterodimer (Tanaka et al., 2007; Fuerer et al., 2014). Now, in eLife, three groups report that they have resolved long-standing questions about the function of Gdf3 by generating mutations in the *gdf3* gene in zebrafish (Bisgrove et al., 2017; Montague and Schier, 2017; Pelliccia et al., 2017).

The proteins in the TGFß family contain two domains – a prodomain and a mature ligand domain – and they are translated in the endoplasmic reticulum, where they form either homodimers or heterodimers (Figure 1). These dimers are then cleaved to yield dimers that contain just the two mature ligand domains (Constam, 2014). After being secreted into the extracellular space, the mature ligand dimers can bind a receptor complex on a neighboring cell and trigger a sequence of events that leads to the expression of genes involved in the development of the mesoderm and endoderm, and in left-right patterning (Figure 2).

**Figure 1.**
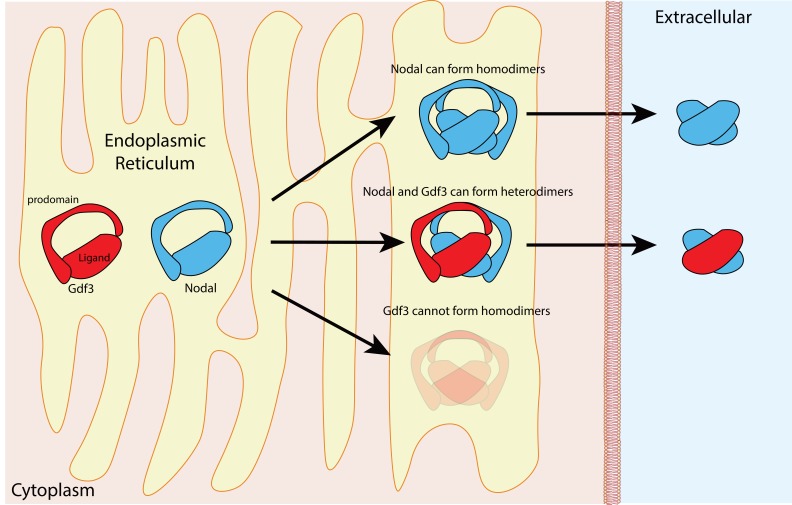
Nodal homodimers and Nodal-Gdf3 heterodimers. Gdf3 (red) and Nodal (blue) both contain an N-terminal prodomain and a mature C-terminal ligand when they are first synthesized in the endoplasmic reticulum. Nodal can form homodimers, and also heterodimers with Gdf3, but Gdf3 is unable to form homodimers. Although cells can form Nodal homodimers, Nodal-Gdf3 heterodimers predominate in signaling (after cleavage in the endoplasmic reticulum and secretion into the extracellular space).

**Figure 2.**
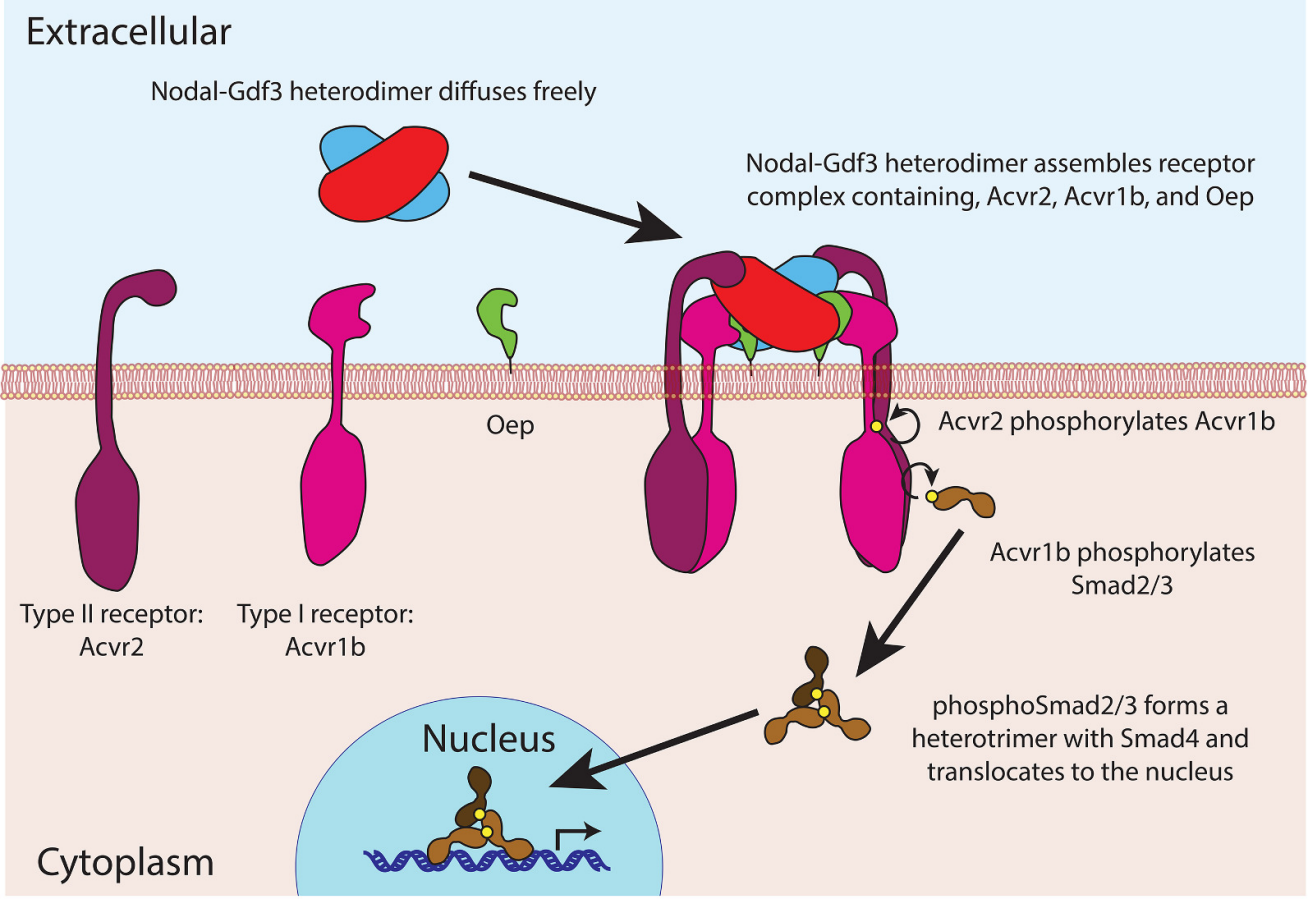
Nodal-Gdf3 signaling in zebrafish. After being secreted, the mature Nodal-Gdf3 heterodimer (red and blue) diffuses through the extracellular space to the surface of the receiving cell, where it binds a co-receptor called Oep (short for one-eyed-pinhead; Gritsman et al., 1999) and two receptors (Acvr1b and Acvr2; Wrana et al., 1994) to assemble a signaling complex. Within this complex, the Acvr2 receptors phosphorylate the Acvr1b receptors, which in turn phosphorylate proteins called Smad2 and Smad3 (Gu et al., 1998; Dubrulle et al., 2015). The phosphorylated Smad2 or Smad3 then forms a heterotrimer with Smad4 and accumulates in the nucleus, where it activates the transcription of various genes. The signaling complex shown here was first suggested by Calvanese et al. (2010).

Perplexingly, Gdf3 is neither cleaved nor secreted when overexpressed (Dale et al., 1989; Tannahill and Melton, 1989), deepening the mystery of its function. However, replacing the prodomain of Gdf3 with the prodomain of a different member of the TGFß family allows it to be cleaved and secreted, after which it is able to specify the mesoderm and endoderm (Dale et al., 1993; Thomsen and Melton, 1993; Kessler and Melton, 1995), but this does not explain why ordinary Gdf3 cannot be cleaved in the first place. The three papers published in eLife have finally illuminated the role of Gdf3.

The groups use different approaches but they all conclude that Nodal and Gdf3 function by forming a heterodimer. The experiments involved creating two types of mutants: *gdf3* zygotic mutants and maternal-zygotic (MZ) *gdf3* mutants. *gdf3* zygotic mutants are deficient in *gdf3* mRNA transcribed from the embryonic genome after fertilization, and MZ-*gdf3* mutants are deficient in both maternal *gdf3* (that is, *gdf3* mRNA and protein made during oogenesis and loaded into the egg before fertilization) and zygotic *gdf3.*

In one paper Brent Bisgrove, Yi-Chu Su and Joseph Yost of the University of Utah showed that Gdf3 acts in parallel, and not upstream or downstream, to Nodal signaling (Bisgrove et al., 2017). They demonstrated that the downstream machinery of Nodal/Gdf3 signaling was functionally intact in the MZ-*gdf3* mutants: moreover, they eliminated the possibility that signaling is lost through the overexpression of a Nodal antagonist called Lefty. Furthermore, Bisgrove et al., and also Tessa Montague and Alexander Schier of Harvard University (Montague and Schier, 2017), showed that Gdf3 and Nodal must be co-expressed in the same cells for signaling to occur. Because maternal Gdf3 is ubiquitously expressed in the embryo, it had not been appreciated in the past that Nodal signaling depends entirely on its co-expression with Gdf3. Using MZ-*gdf3* mutants, both groups found that the only way to rescue the patterning of the mesoderm and endoderm was for *gdf3* and *nodal* to be co-expressed in the same cells.

Montague and Schier delved further into the existence and mechanism of the Nodal-Gdf3 heterodimer, resolving longstanding questions about the secretion of Gdf3 in the process. Through co-immunoprecipitation, they confirmed that Nodal-Gdf3 heterodimers form in the embryo, and they failed to find Gdf3 homodimers. They also demonstrated that the prodomain of Gdf3 cannot be cleaved from the mature ligand without the co-expression of Nodal. Using fluorescent tags they also found that Gdf3 was only secreted when co-expressed with Nodal, and that secreted Gdf3 co-localizes with secreted Nodal. In summary, Montague and Schier demonstrated that the cleavage of Gdf3, the secretion of Gdf3 and Gdf3 signaling activity all depended on the formation of the Nodal-Gdf3 heterodimers.

In the third paper Jose Pelliccia, Granton Jindal and Rebecca Burdine of Princeton University revealed the role of maternal *gdf3* in left-right patterning (Pelliccia et al., 2017). These researchers identified the same loss of mesoderm and endoderm patterning as the other groups, but they were curious about the lack of a left-right patterning phenotype in zygotic *gdf3* mutants. This was surprising because previous *gdf3* inhibition experiments caused left-right patterning defects, and also because zygotic *gdf3* is co-expressed with *nodal* in tissues where Nodal regulates left-right patterning (Zinski et al., 2017)).

Pelliccia et al. performed clever experiments that gave the answer. They found that while a high concentration of *gdf3* mRNA restored left-right asymmetry in the MZ-*gdf3* mutant, progressively lower concentrations revealed left-right asymmetry defects. These results indicate that, surprisingly, maternally expressed *gdf3* mRNA and/or protein persists to post-gastrulation stages of development, when it functions together with Nodal as a Nodal-Gdf3 heterodimer to generate left-right asymmetry in the embryo, obviating the need for zygotic Gdf3 expression.

Together these three papers make a compelling case for the existence and importance of Nodal-Gdf3 heterodimers during early development, but many questions still remain unanswered. Why, for example, are heterodimers required? Montague and Schier propose a model in which the Gdf3 proteins are formed first and wait for Nodal protein to form in the endoplasmic reticulum: the presence of a large population of ready-made Gdf3 monomers then allows the cell to make the Nodal-Gdf3 heterodimers more rapidly than it could make Nodal homodimers. However, this explanation does not account for the increased potency of Nodal-Gdf3 heterodimers compared to Nodal homodimers, which implies that downstream effects could be more important. Perhaps, for example, the asymmetry of the heterodimer leads to more efficient signal transduction through the receptors to phosphorylate Smad2/3 (Figure 2). Interestingly, another member of the TGFß family, Bmp2-Bmp7, also functions exclusively as a heterodimer in patterning dorsal-ventral tissues during the same stages of development that Nodal-Gdf3 heterodimers specify the mesoderm and endoderm (Little and Mullins, 2009).

Altogether, these papers represent a substantial leap forward in our understanding of Nodal and Gdf3 function during early embryonic development and reveal the reigning of TGFß heterodimers in the embryo.
